# Enzymatic characterization and docking simulation of a xylan synthase catalytic subunit, *Setaria viridis* IRX10, using xylotrimer acceptors with distinct fluorescent labels

**DOI:** 10.5511/plantbiotechnology.25.0123a

**Published:** 2025-06-25

**Authors:** Seichi Suzuki, Yasuhiko Kizuka, Bunzo Mikami, Kosei Yamauchi, Takeshi Ishimizu, Shiro Suzuki

**Affiliations:** 1 United Graduate School of Agricultural Science, Gifu University; 2 Graduate School of Natural Sciences and Technologies, Gifu University; 3 Faculty of Applied Biological Sciences, Gifu University; 4 Institute for Glyco-core Research (iGCORE), Gifu University; 5 Research Institute for Sustainable Humanosphere, Kyoto University; 6 Institute of Advanced Energy, Kyoto University; 7 College of Life Sciences, Ritsumeikan University

**Keywords:** arabinoxylan biosynthesis, docking simulation, fluorescent labeling, IRX10, *Setaria viridis*

## Abstract

Arabinoxylan, a major hemicellulose in plant cell walls, particularly in grasses and cereals, plays a crucial role in structural integrity and biological functions, with diverse industrial applications such as food production and prebiotic development. Despite its significance, the molecular mechanism of arabinoxylan biosynthesis remains unclear. Here, we identified and characterized a xylan synthase catalytic subunit, *Setaria viridis* IRregular Xylem 10 (SvIRX10), from a new model plant for C_4_-photosynthetic grasses, *S. viridis* A10.1. Bioinformatic analysis classified SvIRX10 as a glycosyltransferase 47 family member, conserved across various species. Recombinant SvIRX10 expressed in Expi293 cells exhibited xylan synthase activity for all tested xylotrimer (Xyl_3_) acceptors with distinct fluorescent labels. The substrate conversion efficiency for 2-aminobenzoic acid-labeled Xyl_3_ (Xyl_3_-2AA) was highest, but those for other labeled Xyl_3_ were lower. Nevertheless, the elongation efficiencies were comparable among tested acceptors when the xylan chains elongated enough. Structural prediction and docking simulations illustrated most frequently the productive conformations using Xyl_3_-2AA and xylotetraose as ligands. The interactions between the two ligands and the active site were well-conserved, and all ligand units interacted with SvIRX10. These ligand conformations in the active site were similar, but those of other fluorescently labeled Xyl_3_ differed except for the first xylosyl unit at the non-reducing end. Thus, SvIRX10 recognizes at least 4 xylosyl units in the xylan synthetic reaction. Together, these findings provide insights into the enzymatic mechanisms of SvIRX10 and the initiation of xylan elongation, offering potential applications for modifying plant cell walls in biomass utilization and functional food development.

## Introduction

Arabinoxylan is a major hemicellulosic component of plant cell walls in Gramineae species, composed of a β-(1,4)-linked xylan backbone with arabinofuranose side chains at the 2-, 3-, or both hydroxy groups of xylose residues. Monosaccharides like xylose, galactose, and arabinofuranose are occasionally attached to the arabinofuranose side chain. Additionally, the 5-hydroxyl group of 3-*O*-arabinofuranose side chains is often esterified by phenolic acids such as ferulate or *p*-coumarate (Curry et al. 2023; Faik et al. 2014; Rennie and Scheller 2014; Ye and Zhong 2022; York and O’Neill 2008). Arabinoxylan provides structural integrity to plant cell walls, offering pathogen defense and mechanical strength (Freeman et al. 2017). However, its chemical complexity presents challenges for biomass conversion, especially in biofuel production (Smith et al. 2017). Meanwhile, the oligosaccharides function as prebiotics, antioxidants, and human immune enhancers (Suzuki et al. 2023). Thus, understanding arabinoxylan biosynthesis and the enzymes involved is crucial for optimizing industrial applications (Wierzbicki et al. 2019).

Arabinoxylan biosynthesis involves glycosyltransferases (GTs) assembling the xylan backbone and side chains, mainly in the Golgi apparatus. The xylan backbone is synthesized by the xylan synthase complex *in vivo*. In the proposed model of the xylan synthase complex, IRX9, IRX14, and their homologs, which are all classified into the GT43 family, are anchored to the Golgi membrane and assist IRX10 inside the Golgi (Anders et al. 2023; Javaid et al. 2024; Jiang et al. 2016; Zeng et al. 2016). IRX10 and its homologs have been known as catalytic subunits responsible for xylan backbone elongation. *IRX10* was initially identified as a member of the *IRX* group in Arabidopsis (*Arabidopsis thaliana*), with *irx10* mutants showing a slight reduction in xylan content and mild growth defects (Brown et al. 2005). Subsequently, the crucial function of IRX10 in xylan biosynthesis was demonstrated by the loss of both *IRX10* and *IRX10L*, causing a severe reduction of xylan content, abnormal cell wall formation, and stunted growth in Arabidopsis (Brown et al. 2009; Wu et al. 2009). Recombinant Arabidopsis IRX10L showed xylosyltransferase activity, using UDP-xylose as a donor (Urbanowicz et al. 2014). Xylan synthase activities of IRX10 and homologs have been demonstrated to be conserved among multiple species covering algae to land plants, including *Klebsormidium flaccidum*, *Physcomitrella patens*, *Plantago ovata*, Asparagus (*Asparagus officinalis*), and wheat (*Triticum aestivum*) (Jensen et al. 2014, 2018; Jiang et al. 2016; Smith et al. 2022; Zeng et al. 2016). Recently, rice (*Oryza sativa*) IRX10 (OsIRX10) has been shown to play a key role in elongating xylan backbones. The rice genome harbors six homologs of IRX10, all of which contribute to xylan elongation *in vivo* (Wang et al. 2022). These homologs were recently demonstrated to require xylobiose as a minimum acceptor for xylan elongation reactions with different elongation efficiencies (Zhong et al. 2024). Interestingly, OsIRX10 exhibited *de novo* xylan synthetic activity without xylooligosaccharide acceptors. However, this observation could not be demonstrated in the subsequent study, leaving open questions about the xylan initiation mechanism with considerable interest (Curry et al. 2023; Zhong et al. 2024).

While IRX10 and its homologs have been studied in rice, their roles in other grasses remain largely unexplored. *Setaria viridis* has emerged as a model Gramineae plant for functional genomics due to its short life cycle, ease of cultivation, and ability for *Agrobacterium*-mediated genetic manipulation (Brutnell et al. 2010; Mamidi et al. 2020). *S. viridis*, ancestral to foxtail millet (*S. italica*), is more closely related to major C_4_ crops like maize, sorghum, and sugarcane than other Gramineae models like rice and Brachypodium (*Brachypodium distachyon*) (Li and Brutnell 2011). These characteristics render *S. viridis* ideal for studying important traits like C_4_-photosynthesis and drought tolerance related to the cell wall structure.

**Figure 1:**
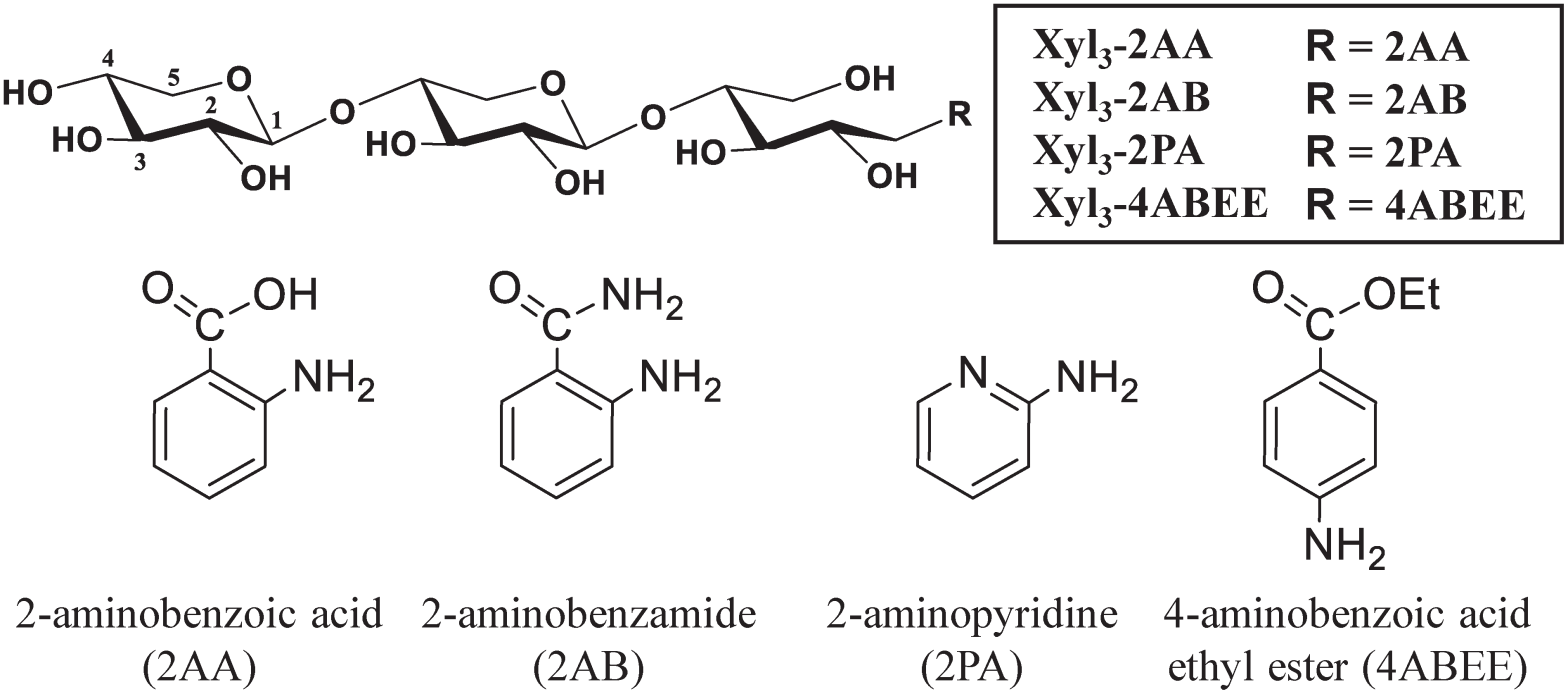
Figure 1. Chemical structures of fluorescently labeled xylotrimers (Xyl_3_) and the four reagents used for fluorescent labeling. Fluorescently labeled Xyl_3_ were prepared by reductive amination of xylotriose with the reagents 2AA, 2AB, 2PA, and 4ABEE.

The appropriate selection of acceptors with degree of polymerization (DP) and fluorescent labels acceptable for carbohydrate-active enzymes (CAZymes), including glycosyltransferases, is essential for the highly sensitive and quantitative assays (Drula et al. 2022). Compared to post-reaction labeling, which may involve multiple reactive components and a complicated clean-up process, using fluorescently labeled substrates provides a straightforward and efficient approach (Wagner and Pesnot 2010). However, fluorescent labels can influence enzyme activity in diverse ways. For example, labeled sugars have been shown to interfere with substrate recognition, inhibiting bovine α-L-fucosidase by preventing proper binding (O’Flaherty et al. 2017). Conversely, fluorescent labeling enhanced catalytic efficiency in assays for xyloglucan endotransglycosylase from Nasturtium (*Tropaeolum majus*) seeds (Kosík et al. 2011). In earlier works, 2AA- and 2-aminobenzamide (2AB)-labeled xylooligosaccharides with DP ranging from 4 to 6 were used as fluorescently labeled acceptors in xylan synthase assays (Jensen et al. 2014, 2018; Urbanowicz et al. 2014; Zhong et al. 2024). However, as mentioned above, IRX10 recognizes unlabeled xylooligosaccharides as short as DP2, establishing it as the minimum length required for activity (Urbanowicz et al. 2014; Zhong et al. 2024). Moreover, 2-aminopyridine (2PA) and 4-aminobenzoic acid ethyl ester (4ABEE) are also well-known for labeling oligosaccharides in various glycosyltransferase assays (Akita et al. 2002; Takahashi et al. 2024; Urahara et al. 2004). Accordingly, the quantitative measurement of xylan synthase activity using various fluorescently labeled DP3 substrates, where the two cyclic xylosyl residues are at the non-reducing end (Figure 1), may provide new insights into the onset mechanism of the xylan chain elongation in the xylan synthetic reaction.

In this study, we identified an IRX10 ortholog, SvIRX10, in the genome-decoded *S. viridis* A10.1 and expressed it as a recombinant protein. We quantitatively measured the xylan chain elongation activity using Xyl_3_ acceptors with four distinct fluorescent labels. Utilizing bioinformatics, biochemical assays, and docking simulations, we elucidated the biochemical function of SvIRX10 and gained insight into the molecular mechanism of xylan chain elongation.

## Materials and methods

### Bioinformatic analysis

The amino acid sequences of *S. viridis* and those of other GT47 family members were retrieved from Phytozome v13 (Goodstein et al. 2012) and the NCBI database (https://www.ncbi.nlm.nih.gov/). Amino acid alignment and phylogenetic analysis of the GT47 family proteins were carried out using the maximum likelihood method in MEGA11 software (Tamura et al. 2021). Prediction of transmembrane regions, signal peptides, and domain structures was conducted via InterPro protein family database (Blum et al. 2021). Gene expression data of *S. viridis* *IRX10* homologs in various tissues were obtained from Phytozome v13.

### Plant materials and construction of the expression vector

The seeds of *S. viridis* A10.1 (Mamidi et al. 2020), kindly provided by Drs. Kiba and Sakakibara, Nagoya Univ., were sown on peat moss-based soil (TACHIKAWA HEIWA NOUEN, Toyohashi, Japan) and grown at 23°C on a 16 h-light/8 h-dark cycle in a growth chamber. After 6 weeks, the total RNA was extracted from the culm using ISOGEN reagent (Nippon Gene, Tokyo, Japan). The cDNA was synthesized from the RNA using a reverse-transcription kit (Takara Bio, Kusatsu, Japan), and the region excluding the signal sequence and transmembrane domain (amino acids: 28–415) of *S. viridis* IRX10 was amplified using primers (Supplementary Table S1). The PCR fragment was subcloned into the pGEn2-DEST expression vector (Urbanowicz et al. 2014). The details of the expression construct and primer information are described in Supplementary Table S1.

### Expression of recombinant SvIRX10

Recombinant SvIRX10 was produced via a heterologous protein expression system using Human Embryonic Kidney (HEK) Expi293 cells (Thermo Fisher Scientific, Waltham, MA, USA). The cells were transfected with the expression vector using the GMEP transfection system (GMEP, Kurume, Japan). Three days after transfection, the recombinant proteins secreted into the culture medium were purified using Ni Sepharose 6 Fast Flow beads (Cytiva, Tokyo, Japan). Then, the Ni beads with bound proteins were washed with 50 mM HEPES-NaOH buffer (pH 6.8) containing 20 mM imidazole. After washing, the proteins were eluted with the same buffer containing 300 mM imidazole and desalted with 50 mM HEPES-NaOH buffer (pH 6.8). The molecular weight and purity of the proteins were analyzed by sodium dodecyl sulfate-polyacrylamide gel electrophoresis (SDS-PAGE), followed by Coomassie Brilliant Blue staining.

### Xylan synthase activity assay

The 1,4-β-D-xylooligosaccharides (Xyl_3_, xylotriose; Xyl_4_, xylotetraose; Xyl_5_, xylopentaose; Xyl_6_, xylohexaose) and UDP-xylose were purchased from Megazyme (Wicklow, Ireland) and CarboSource Services (Athens, GA, USA). Xylooligosaccharides (Xyl_3_ to Xyl_6_) were fluorescently labeled at the reducing end with 2AA, 2AB, 4ABEE, and 2PA as previously described (Akita et al. 2002; Ishii et al. 2002; Takahashi et al. 2024). The chemical structures of the four fluorescent reagents are shown in Figure 1. In all assays, fluorescently labeled Xyl_3_ was used as the substrate, and labeled Xyl_4_ to Xyl_6_ were used as authentic standards for enzymatic products. The standard reaction mixture (50 µl) consisted of 5 µg of recombinant SvIRX10, 5 mM MgCl_2_, 1 mM UDP-xylose, 0.02 mM Xyl_3_-2AA, and 0.48 mM unlabeled Xyl_3_ in 50 mM HEPES-NaOH buffer (pH 6.8). The unlabeled Xyl_3_ was added to ensure sufficient total substrate concentration while not interfering with evaluating the effects of distinct fluorescently labeled acceptors (Jensen et al. 2014). The mixture was incubated at 25°C for the indicated time and subsequently heated at 95°C for 5 min to stop the reaction. Similar enzymatic assays were conducted using other labeled acceptors (Xyl_3_-2AB, Xyl_3_-4ABEE, and Xyl_3_-2PA) instead of Xyl_3_-2AA. All quantitative enzymatic reactions were performed in triplicate. The reaction products filtered through a 0.45 µm pore-sized membrane were separated by normal-phase high-performance liquid chromatography (NP-HPLC) using a JASCO EXTREMA LC-4000 series with an FP-4020 Fluorescence Detector (JASCO Corporation, Tokyo, Japan) equipped with a Shim-pack GIST Amide column (250 mm×4.6 mm; Shimadzu, Kyoto, Japan) at the flow rate of 1.0 ml min^−1^. Solvents A and B were water containing 0.1% formate and 100% acetonitrile (v/v), respectively. In separating 2AA, 2AB, and 4ABEE derivatives, the following gradient elution program was used: A : B=70 : 30 for 10 min, then linear change to A : B=50 : 50 for 20 min. In separating 2PA derivatives, A : B=65 : 35 for 7 min, then linear change to A : B=50 : 50 for 5 min, and kept at A : B=35 : 65 for 18 min was used. The fluorescent labels were detected using the following conditions: 2AA and 2AB, 330-nm excitation and 420-nm emission; 4ABEE, 305-nm excitation and 360-nm emission; 2PA, 320-nm excitation and 400-nm emission. Quantitative evaluation was based on peak areas calculated using ChromNAV Ver.2 (JASCO Corporation).

### Prediction of the three-dimensional structure of SvIRX10 and docking simulation

The 3D structure of SvIRX10 (amino acids: 28–415) was predicted using ColabFold (Mirdita et al. 2022). The five models were generated with default settings, and the model with the highest predicted local distance difference test (pLDDT) score was used for further analysis. Docking simulations were carried out using SwissDock (Bugnon et al. 2024), applying the Attracting Cavities 2.0 algorithm (Röhrig et al. 2023) to evaluate interactions between SvIRX10 and UDP, xylotetraose, cellotetraose, or four fluorescently labeled Xyl_3_. All fluorescently labeled ligands were drawn in ChemDraw (Revvity Software, Yokohama, Japan) and optimized in Chem3D (Revvity Software) through energy minimization and molecular dynamics simulations to refine the structures and the other ligands were obtained from the PubChem database (https://pubchem.ncbi.nlm.nih.gov). The docking simulation was conducted with a particular focus on the predicted active site of SvIRX10, which was inferred from the known UDP-binding region in Exostosin (EXT) 1 (PDB entry: 7UQY; Li et al. 2023). All other settings were set to default values. The output structures with the lowest binding energies and appropriate conformations were used to evaluate interactions between the protein and ligands (Supplementary Table S2). Visualization and further analysis of the binding interactions were performed using UCSF ChimeraX (Goddard et al. 2018) and CCP4 (Winn et al. 2011).

## Results and discussion

### Bioinformatic analysis of SvIRX10 and its homologs in *S. viridis*

**Figure 2:**
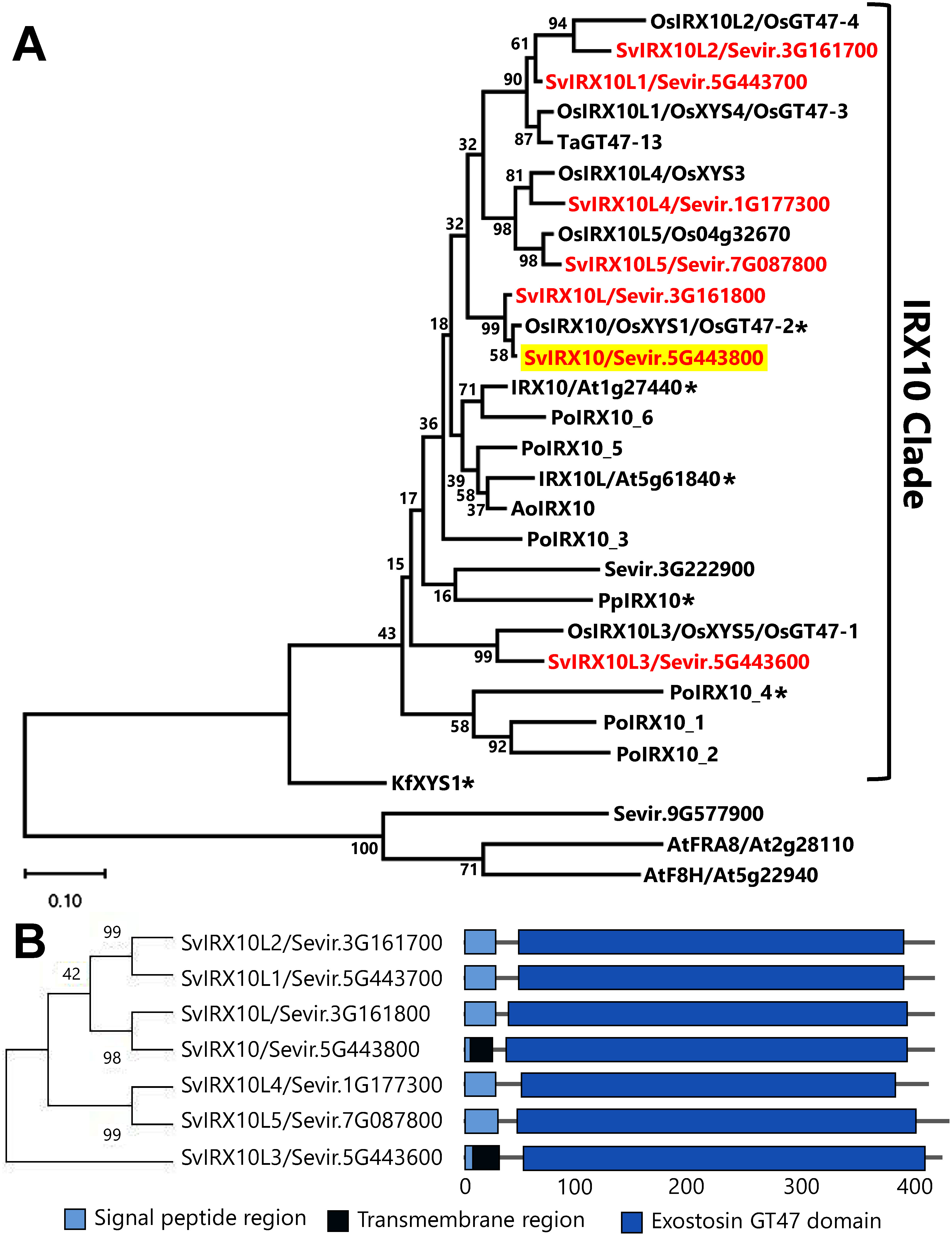
Figure 2. Bioinformatic analysis of GT47 family proteins in *Setaria viridis*. (A) A phylogenetic tree of GT47 family proteins, including rice (Os), wheat (Ta), Asparagus (Ao), moss (Pp), Psyllium (Po), *K. flaccidum* (Kf), Arabidopsis (At), and *S. viridis* (Sv) IRX10-like proteins. SvIRX10, characterized in this report, is highlighted in yellow. The asterisks indicate the proteins of which *in vitro* xylan synthase activities have been reported. AtFRA8 and AtF8H are used as outgroups. Node numbers are bootstrap values (percentages) from 1,000 replicates. (B) A phylogenetic tree and domain structure of SvIRX10 and its homologs.

Biochemical analysis of IRX10 and its homologs from rice and the other species has classified these proteins into the IRX10 clade within the GT47 family (Javaid et al. 2024; Zhang et al. 2023; Zhong et al. 2024). Using rice IRX10 homologs as queries, we performed a BLASTP search and identified seven IRX10 homologs in the *S. viridis* genome (Figure 2A). This number exceeds the two found in Arabidopsis (IRX10 and IRX10L) and the six in *P. ovata* (PoIRX10_1–6) (Jensen et al. 2014; Urbanowicz et al. 2014). In contrast, *P. patens* (PpIRX10) and *K. flaccidum* (KfIRX10) contain only one homolog (Jensen et al. 2014, 2018), indicating that the evolution of xylan biosynthesis commenced before the advent of land plants. The variation of IRX10 homologs suggests functional diversification linked to cell wall complexity across plant lineages. Gramineae plants, including *S. viridis*, have high levels of arabinoxylan in both primary and secondary walls, contributing to mechanical strength and environmental adaptation.

According to predictions by InterPro, all IRX10 homologs in *S. viridis* possessed a signal peptide. Additionally, SvIRX10 and SvIRX10L3 were suggested to have a transmembrane domain (Figure 2B). The presence of signal peptides in all homologs and additional transmembrane domains in a few homologs was observed in OsIRX10 and its homologs in rice (Wang et al. 2022). The prediction of signal peptides in all IRX10 homologs of *S. viridis* suggested that they are not cytosolic proteins. It is noteworthy that the signal peptides alone are not likely responsible for the Golgi localization of glycosyltransferases, and additional factors, such as protein-protein interactions and specific transport mechanisms, may also be required (Javaid et al. 2024; Zeng et al. 2016). On the other hand, the prediction of transmembrane domains may support the idea that SvIRX10 and SvIRX10L3 are anchored in the Golgi membrane. Indeed, the wheat IRX10 homolog TaGT47-13 possessed both a signal peptide and a transmembrane domain and was anchored in the endoplasmic reticulum and the Golgi membrane (Jiang et al. 2016). In contrast, IRX10 homologs lacking transmembrane domains may be supported by IRX9, IRX14, and their homologs within the Golgi lumen as illustrated by AoIRX10 in Asparagus (Zeng et al. 2016). Recently, rice GT43 and GT47 families were demonstrated to form specific protein-protein interactions and to comprise the central cores of xylan synthase complexes. The central core was initially formed in the endoplasmic reticulum and accumulated in the Golgi, emphasizing the importance of proper glycosyltransferase localization and coordination in xylan biosynthesis (Javaid et al. 2024). Nevertheless, the signal peptides may act as a cryptic transmembrane domain to the Golgi membrane (Jiang et al. 2016). Therefore, the identification of signal peptides/transmembrane domains in the N-termini of IRX10 and its homologs is challenging and awaits further investigation.

Differential gene expression across tissues (Supplementary Figure S1) suggests distinct roles of SvIRX10 and homologs in various developmental and physiological events in *S. viridis*. For example, *SvIRX10* shows ubiquitous expression across all tissues, suggesting its fundamental involvement in xylan biosynthesis. In contrast, *SvIRX10L5* is predominantly expressed in leaves and shoots, the tissues rich in primary cell walls, suggesting a specialization for primary cell wall biosynthesis in grasses. Furthermore, recent studies on grass xylan structural variation have highlighted the presence of distinct xylan types, such as arabinoxylan with evenly distributed arabinose substitutions (AXe), glucuronoarabinoxylan with clustered glucuronic acid modifications (GAXc), and highly substituted glucuronoarabinoxylan (hsGAX), which may reflect functional specialization in their interaction with cellulose and lignin (Tryfona et al. 2023). These findings suggest that the diversified IRX10 homologs may be associated with the structural variation of xylans in grasses. On the other hand, Javaid et al. (2024) recently proposed that OsGT47-1 (alias of OsIRX10L3 and OsXYS5) and OsGT47-3 (alias of OsIRX10L1 and OsXYS4) function in xylan biosynthesis in rice starchy endosperm tissues and vegetative primary cell walls, whereas OsGT47-2 (alias of OsIRX10 and OsXYS1) and OsGT47-4 (alias of OsIRX10L2) act in aleurone cells of rice seeds, which have both primary and secondary cell walls. OsIRX10/OsXYS1/OsGT47-2 was also reported to be associated with xylan biosynthesis in lignified pit borders, where secondary cell walls are augmented in rice plants (Wang et al. 2022). Our phylogenetic tree suggested the orthologous relationship between SvIRX10 and OsIRX10/OsXYS1/OsGT47-2 (Figure 2A), implying a similar function shared by these two putative orthologs. Collectively, due to its widespread expression and potential functional importance, *SvIRX10* (*Sevir.5G443800*) was selected for subsequent biochemical studies to elucidate its role in arabinoxylan biosynthesis.

### Recombinant SvIRX10 protein has xylan synthase activity

**Figure 3:**
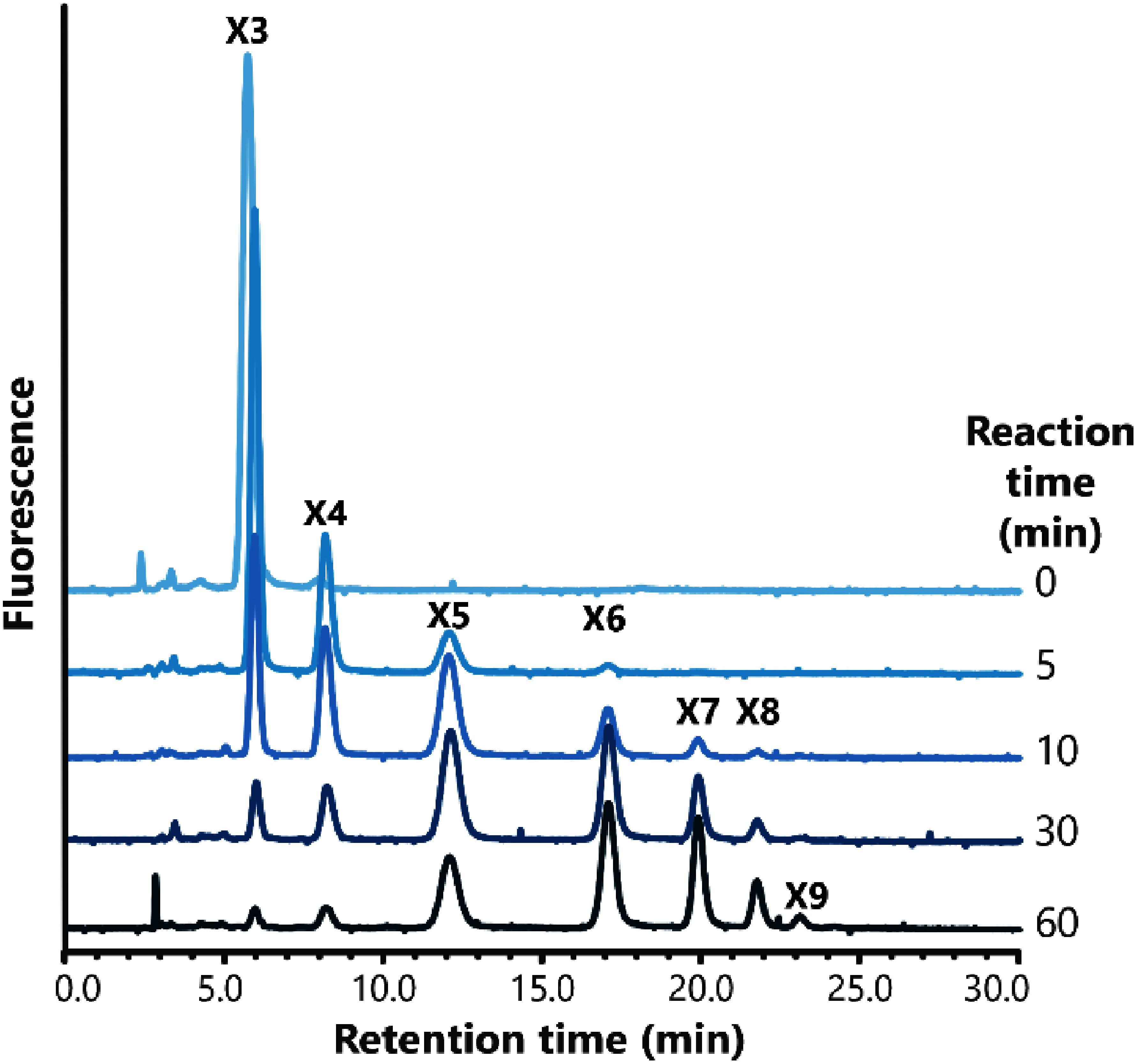
Figure 3. Time course of xylan chain elongation by recombinant SvIRX10. Recombinant SvIRX10 was incubated in HEPES-NaOH buffer (pH 6.8) with UDP-xylose, Xyl_3_-2AA, unlabeled Xyl_3_, and MgCl_2_ for the indicated reaction time. The reaction products were analyzed on NP-HPLC. The numbers of X3 to X9 represent the DP of xylooligomers.

To investigate the biochemical function of SvIRX10, the recombinant protein was expressed using a heterologous Expi293 cell system. The protein was successfully secreted into the culture media and purified as an 8×His-tagged GFP fusion. Xylan synthase activity was quantified by incubating the recombinant SvIRX10 with Xyl_3_-2AA, unlabeled Xyl_3_, and UDP-xylose, but no activity was detected when the enzyme was heat-inactivated (Supplementary Figure S2). NP-HPLC analysis revealed time-dependent elongation of xylooligosaccharides, indicating that SvIRX10 can continuously transfer xylosyl residues to the acceptor (Figure 3). Remarkably, one to three xylosyl units were attached within just 5 min, demonstrating the rapid initiation of xylan elongation. The extension of incubation time to 60 min resulted in the production of DP9 xylosyl units, contrasting the earlier recombinant IRX10 assay results: Arabidopsis IRX10L expressed in HEK293 cells elongated xylosyl units from DP6 to DP13 in 16 h; KfXYS1 expressed in yeast afforded xylosyl units from DP4 to DP13 in 4 h; and OsXYS1 expressed in HEK293 cells gave xylosyl residues from DP4 to DP11 in 16 h (Jensen et al. 2018; Urbanowicz et al. 2014; Zhong et al. 2024). Together, our results demonstrated that the assay for 60 min is sufficient to obtain results analogous to those of the previous reports, underscoring our highly active recombinant enzyme and sensitive assay system.

### Difference in SvIRX10 xylan synthase activity using Xyl_3_ with distinct fluorescent labels

**Figure 4:**
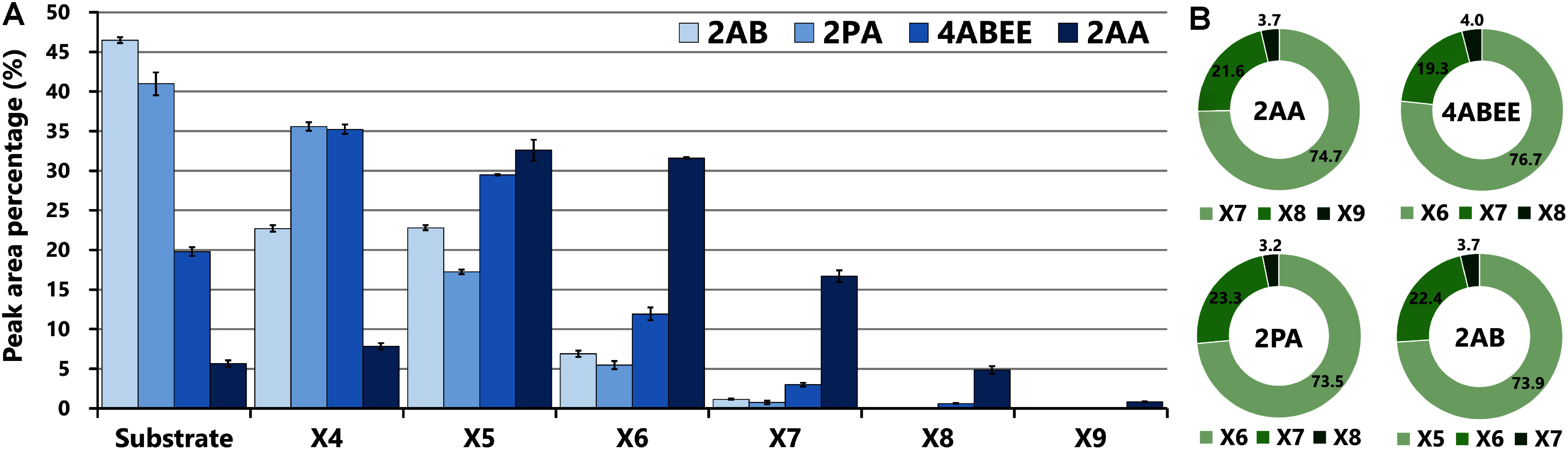
Figure 4. Effect of Xyl_3_ acceptors with distinct fluorescent labels on the xylan synthase activity of SvIRX10. (A) The differences in xylan synthase activity by SvIRX10 based on substrate consumption and product formation measured by NP-HPLC. Each assay was performed with SvIRX10 using four fluorescently labeled Xyl_3_ (Xyl_3_-2AA, -2AB, -4ABEE, and -2PA) for 30 min. A bar graph indicates the average percentage of peak areas in the NP-HPLC chromatograms of triplicated assays. The numbers of X4 to X9 indicate the DP of xylooligomers. Error bars represent SD. (B) Ratios of the three longest elongated products with fluorescently labeled Xyl_3_ obtained after the reactions. The sum of percentages of the three longest elongated products (e.g., X7, X8, and X9 for 2AA) shown in Figure 4A were set to 100, and then the ratios were calculated.

As demonstrated in previous studies, DP2 (xylobiose) is the minimum length of acceptors necessary for IRX10 activity assay (Urbanowicz et al. 2014; Zhong et al. 2024). However, the quantitative analysis of the initiation reaction of xylan elongation is still lacking. Moreover, various IRX10 assays have employed labeled substrates from DP4 to DP6, which did not afford information about the onset mechanism of xylan chain elongation. To investigate the initiation of xylan chain elongation with appropriate fluorescent labels, we prepared Xyl_3_ acceptors labeled with four distinct fluorescent labels, i.e., 2AA, 2AB, 4ABEE, and 2PA, and quantitated the xylan synthase activity of SvIRX10. As a result, all fluorescently labeled Xyl_3_ were used in the reaction, but the enzymatic activity of SvIRX10 varied significantly depending on the fluorescent labels of the acceptors (Figure 4A). The substrate conversion efficiency at the initial stage of xylan elongation was calculated based on the percentage of substrate consumed during the enzymatic reaction: 2AA-labeled acceptors exhibited the highest efficiency, reaching 94.4%, followed by 4ABEE-labeled acceptors with 79.8%. In contrast, 2AB- and 2PA-labeled acceptors showed slower reaction rates, with conversion efficiencies of 53.5% and 59.1%, respectively. Conversely, as the xylan chain length reached DP5 (X5) or beyond, the influence of the fluorescent labels became less pronounced. The distribution of elongated products with the first to third longest chain lengths was comparable regardless of the fluorescent label types (Figure 4B). Additionally, our results demonstrated that 4ABEE- and 2PA-labeled Xyl_3_ were acceptable for recombinant SvIRX10 in addition to 2AA- and 2AB-labeled acceptors, the fluorescent labels used in the previous reports (Jensen et al. 2014, 2018; Urbanowicz et al. 2014; Zhong et al. 2024). The reactivity of fluorescently labeled Xyl_3_, in which two cyclic xylosyl residues are situated in the non-reducing end, corroborated the observation that unlabeled xylobiose (DP2; X2) is the minimum length-acceptor required for IRX10 activities (Urbanowicz et al. 2014; Zhong et al. 2024), suggesting that the process of substrate recognition in the initial stage of xylan elongation by IRX10 and its homologs is conserved across species. Taken together, fluorescent molecules labeled with Xyl_3_ significantly affected the substrate conversion efficiency at the initial stages of xylan elongation (e.g., conversions of X3 to X4 and X4 to X5). However, as the chain length increases, it is likely that the impact of the labels diminishes, exhibiting the comparable catalytic activities of SvIRX10 regardless of the label types.

### Structural prediction and docking simulations of SvIRX10

**Figure 5:**
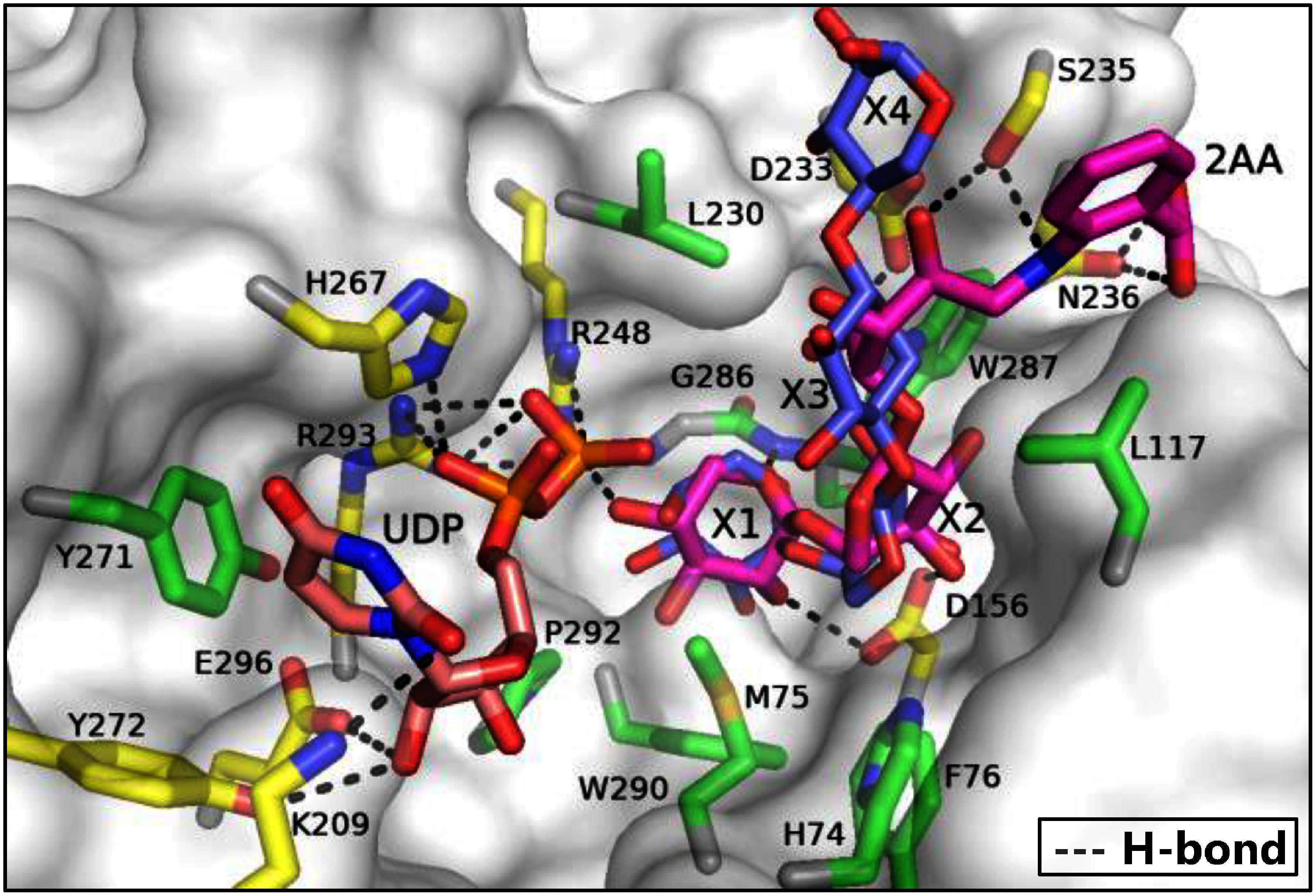
Figure 5. The predicted 3D model of SvIRX10 complexed with UDP and Xyl_3_-2AA. Hydrogen bonds of SvIRX10 (amino acids: 28–415) with UDP and Xyl_3_-2AA are shown in black dashed lines. The amino acid residues involved in the hydrogen bonds and the C-C contacts are highlighted in yellow and green carbons, respectively. Xyl_3_-2AA (magenta C, red O, and blue N) and the protein surface (light gray) are shown. For comparison, a native acceptor, unlabeled xylotetraose (pale blue C and red O), was overlaid. The non-reducing end xylosyl residues of Xyl_3_-2AA and xylotetraose are labeled as X1.

To gain insight into the diverse activities of SvIRX10 using the Xyl_3_ acceptors with distinct fluorescent labels during the early stages of xylan elongation, we predicted the 3D structure of SvIRX10. Then, we performed docking simulations with UDP and Xyl_3_ with four distinct fluorescent labels (2AA, 2AB, 4ABEE, and 2PA), an unlabeled native acceptor, xylotetraose, as a positive control, and cellotetraose as a negative control to evaluate the specificities of the SvIRX10 xylan synthase activity. First, the 3D model of SvIRX10 (amino acids: 28–415) was generated using ColabFold, yielding a highly reliable structure with a pLDDT score of 94.5 (Supplementary Figure S3). This predicted structure was then compared with the experimentally resolved 3D structure of EXT1 GT-B, a glycosyltransferase from the GT47 family. Structural alignment revealed a high similarity between SvIRX10 and EXT1 GT-B, particularly in the active site region, as indicated by a root mean square deviation (RMSD) of 1.263 Å based on 115 pruned and aligned Cα atoms. Next, UDP was docked into the active site of SvIRX10, resulting in its binding to E296 (Figure 5). This glutamate is highly conserved among GT47 family proteins, including EXT1, EXT2, and EXTL3 (Li et al. 2023; Wilson et al. 2022; Supplementary Figure S4). To evaluate the binding energy and compare the interactions between fluorescently labeled Xyl_3_ acceptors and SvIRX10, we simulated the non-reducing end of fluorescently labeled Xyl_3_ to fit deeply into the active site using SwissDock, similarly to complexing a trisaccharide GlcNAc-GlcA-GlcNAc and UDP-GlcA with EXT1 GT-B (Li et al. 2023; Supplementary Figure S5). The ten highest-ranked binding models, along with the corresponding binding energies and docking positions, were obtained (Supplementary Table S2). The lowest binding energies at the correct binding site of the ligand were observed as follows: −8.57, −8.27, −8.18, and −8.04 kcal mol^−1^ for Xyl_3_-2AA, Xyl_3_-4ABEE, Xyl_3_-2AB, and Xyl_3_-2PA, respectively, and all of them retained productive conformations. Similarly, the native acceptor, unlabeled xylotetraose, exhibited a binding energy of −8.25 kcal mol^−1^, comparable with those of the four labeled acceptors. In contrast, docking simulations revealed that cellotetraose frequently overlapped with the UDP-binding site or adopted inverted orientations, preventing proper alignment within the active site. This misalignment may account for the lack of enzymatic activity of Arabidopsis IRX10L for cellopentaose (Urbanowicz et al. 2014). Nevertheless, among the four fluorescently labeled Xyl_3_ acceptors, Xyl_3_-2AA exhibited the lowest binding energy, supporting its superior xylan synthase activity observed in the early stages of the enzymatic reaction. Regarding spatial positioning, the frequency of UDP misalignment (*hindered*) was lowest for Xyl_3_-2AA, followed by Xyl_3_-4ABEE, Xyl_3_-2PA, and Xyl_3_-2AB (Supplementary Table S2). The high frequency of misalignment observed for Xyl_3_-2AB may correlate with its lower enzymatic activity, as such configurations hinder effective substrate positioning and catalysis. Together, these results highlight the importance of both binding energy and efficient spatial alignment of substrates, with Xyl_3_-2AA showing the best overall compatibility with the active site.

### Interactions between Xyl_3_ with distinct fluorescent labels and SvIRX10 in the active site

To extend the insights from the docking simulations, we focused on the spatial interactions between the ligands and SvIRX10, examining how specific hydrogen bonding and ligand conformations were involved. The docking simulation revealed that Xyl_3_-2AA, the substrate with the highest enzymatic activity, formed interactions with key amino acid residues similar to those of the native acceptor, Xyl_4_ in the active site of SvIRX10 (Figure 5). Comparison of the predicted hydrogen bonds and C-C contacts between SvIRX10/Xyl_4_ and SvIRX10/Xyl_3_-2AA docked models suggested that interactions with the ligands at the active site are mostly shared between Xyl_3_-2AA and xylotetraose, and the conformations of the 2AA label of Xyl_3_-2AA and the X4 residue of xylotetraose differed only slightly (Figure 5; Supplementary Table S3).

On the other hand, the representative conformations of the other labeled Xyl_3_ substrates, Xyl_3_-2PA, Xyl_3_-4ABEE, and Xyl_3_-2AB with the lowest binding energies, differed in the arrangement of residues beyond X1 compared to those of Xyl_3_-2AA and xylotetraose (Supplementary Figure S5). Additionally, the labels in Xyl_3_-4ABEE and Xyl_3_-2PA were positioned differently in the second-top-ranked model with slightly higher binding energies (Supplementary Table S2), implying that the label positions were easily changeable. Nevertheless, the X1 residues in all labeled Xyl_3_ substrates were positioned similarly to that of xylotetraose (Supplementary Figure S5). This suggests that the core recognition of the non-reducing end xylosyl unit requires the minimum enzymatic activity for these substrates. Moreover, the distinct positioning of the X2 and X3 residues in Xyl_3_-2AB, Xyl_3_-4ABEE, and Xyl_3_-2PA within the active site could explain their lower enzymatic activities. Structural deviations in these residues might affect their interactions with the catalytic amino acids, potentially influencing substrate binding affinity and overall reaction efficiency (Supplementary Figure S5). Collectively, the appropriate conformations and interactions of the Xyl_3_ acceptors with the surrounding amino acids were essential for the efficient transfer of xylosyl residues to the acceptors. However, the conformational differences in the X2 and the subsequent residues may be more or less acceptable for xylan chain elongation.

Our view further predicted that the fluorescent labels of sufficiently longer acceptors may not impede xylan synthase activity. Indeed, the attachment of 6 to 10 xylosyl residues to Xyl_4_ (Jensen et al. 2014) and Xyl_6_ (Urbanowicz et al. 2014) with 2AB labels in xylan synthase reaction indicated that longer chain length minimizes the impact of the label on enzyme function. In our docking simulations, all residues of Xyl_3_-2AA (including 2AA label) and xylotetraose interact with the amino acids of SvIRX10. Accordingly, SvIRX10 could recognize at least 4 xylosyl residues in xylan chain elongation, and the residues beyond may not be associated with this reaction. This notion implies that the fluorescent labels attached to Xyl_5_, which have 4 cyclic xylosyl units at the non-reducing end, may not affect the xylan synthase activity.

In conclusion, this study provides insights into the enzymatic mechanism of the grass IRX10 ortholog, SvIRX10 from *S. viridis*. By analyzing Xyl_3_ acceptors with distinct fluorescent labels, we demonstrated how substrate structure and active-site interactions influence the xylan synthase activity of SvIRX10. The ability to recognize 4 xylosyl units and the allowable conformations in the active site delineates the molecular mechanism of xylan synthesis. These findings contribute to understanding the molecular basis of xylan biosynthesis in plants and its potential applications in plant cell wall modification and biomass utilization.

## Abbreviations

2AA: 2-aminobenzoic acid
2AB: 2-aminobenzamide
2PA: 2-aminopyridine
4ABEE: 4-aminobenzoic acid ethyl ester
DP: degree of polymerization
EXT: exostosin
GT: glycosyltransferase
HEK: human embryonic kidney
IRX10: irregular xylem 10
NP-HPLC: normal-phase high-performance liquid chromatography
pLDDT: predicted local distance difference test
RMSD: root mean square deviation
SDS-PAGE: sodium dodecyl sulfate-polyacrylamide gel electrophoresis

## Accession numbers

The GenBank accession number and locus identifier for *SvIRX10* are LC851452 and Sevir.5G443800, respectively.

## Description of supplementary files

Supplementary Table S1. List of PCR primers.

Supplementary Table S2. Simulated scores of SwissDock.

Supplementary Table S3. Interactions between SvIRX10 and ligands (UDP, Xyl_3_-2AA, and xylotetraose) in the docking simulations.

Supplementary Figure S1. Relative gene expression levels of *SvIRX10* homologs in different tissues of *S. viridis*.

Supplementary Figure S2. Detection of xylan synthase activity by recombinant SvIRX10.

Supplementary Figure S3. Predicted model of the SvIRX10 catalytic domain using ColabFold.

Supplementary Figure S4. Partial sequence alignment of GT47 IRX10 clade members with *Homo sapiens* EXT1, EXT2, and EXTL3.

Supplementary Figure S5. Docking simulations of SvIRX10 with fluorescently labeled Xyl_3_ and unlabeled xylotetraose.
