# Supplementary material for: Enzymatic characterization and docking simulation of a xylan synthase catalytic subunit, Setaria viridis IRX10, using xylotrimer acceptors with distinct fluorescent labels
Source: Plant Biotechnol (Tokyo). 2025 Jun 25;42(2):121–9. doi: 10.5511/plantbiotechnology.25.0123a (PMC12235428; doi:10.5511/plantbiotechnology.25.0123a)
Supplement: Supplementary Data [file plantbiotechnology-42-2-25.0123a-s001.pdf]

Supplementary Information

Enzymatic characterization and docking simulation of a xylan synthase catalytic subunit, *Setaria viridis* IRX10, using xylotrimer acceptors with distinct fluorescent labels

Seichi Suzuki, Yasuhiko Kizuka, Bunzo Mikami, Kosei Yamauchi, Takeshi Ishimizu, Shiro Suzuki*

*Corresponding author:

Shiro Suzuki, suzuki.shiro.n5@f.gifu-u.ac.jp

The Supplementary information includes:

Supplementary Table S1 to S3

Supplementary Figure S1 to S5

Supplementary Table S1. List of PCR primers

Oligo name	Orientation	Sequence (5' to 3')	Purpose
SvIRX10_aa23-F	Sense	GCGGTGCAGCAGGGCCACCAGA	cDNA cloning of *SvIRX10**
SvIRX10_aa23-R	Antisense	CTACCAAGGCTTCAGGTCACCCGGTGGTC	cDNA cloning of *SvIRX10**
SvIRX10_pDONR-F	Sense	AACTTGTACTTTCAAGGCCACCAGACAGAGAGGATCTCAGGAAGTGCT	The 1st PCR to subclone into pDONR221**
SvIRX10_pDONR-R	Antisense	ACAAGAAAGCTGGGTCCTACCAAGGCTTCAGGTCACCCGGTGGT	The 1st PCR to subclone into pDONR221**
*att*B_Adapter-F	Sense	GGGGACAAGTTTGTACAAAAAAGCAGGCTCTGAAAACTTGTACTTTCAAGGC	The 2nd PCR to subclone into pDONR221***
*att*B_Adapter-R	Antisense	GGGGACCACTTTGTACAAGAAAGCTGGGTC	The 2nd PCR to subclone into pDONR221***

* SvIRX10 (amino acids: 23–415), excluding the signal peptide/transmembrane domain, was PCR-amplified using cDNA synthesized from total RNA of the *S. viridis* culm as a template and then ligated into the pCR-Blunt II-TOPO vector (Thermo Fisher Scientific, Waltham, MA, USA) to afford pCR-Blunt II-TOPO-SvIRX10.

** The 1st PCR to amplify part of SvIRX10 (amino acids: 28–415) using pCR-Blunt II-TOPO-SvIRX10 as a template.

*** The 2nd PCR to add the *att*B adapter region using the 1st PCR product as a template, which allows the subcloning of the product into pDONR221 (Thermo Fisher Scientific), an entry vector for the Gateway cloning system (Thermo Fisher Scientific).

Supplementary Table S2. Simulated scores of SwissDock

Ligand	Xyl$_3$-2AA				Xyl$_3$-4ABEE				Xyl$_3$-2PA			
		Position				Position				Position		
	Binding energy (kcal mol^{-1})	UDP binding site	Non-reducing end	Direction of 4th residue	Binding energy (kcal mol^{-1})	UDP binding site	Non-reducing end	Direction of 4th residue	Binding energy (kcal mol^{-1})	UDP binding site	Non-reducing end	Direction of 4th residue
1	**-8.57**	**Available**	**Correct**	**Middle**[d]	**-8.27**	**Available**	**Correct**	**Right**[d]	**-8.04**	**Available**	**Correct**	**Right**
2	-8.37	Available	Correct	Middle	-8.21	Available	Correct	Middle	-8.00	Available	Correct	Left[d]
3	-8.35	Available	Slided[b]	—	-8.08	Hindered	Slided	—	-7.93	Available	Slided	—
4	-8.27	Hindered[a]	Inverted[c]	—	-7.92	Hindered	Inverted	—	-7.87	Hindered	Correct	—
5	-8.18	Hindered	Slided	—	-7.91	Hindered	Correct	—	-7.82	Hindered	Slided	—
6	-8.17	Available	Slided	—	-7.69	Hindered	Correct	—	-7.81	Hindered	Correct	—
7	-8.15	Available	Slided	—	-7.59	Available	Correct	Right	-7.79	Hindered	Inverted	—
8	-8.12	Available	Slided	—	-7.58	Available	Correct	Right	-7.74	Available	Correct	Right
9	-8.11	Hindered	Inverted	—	-7.55	Available	Correct	Right	-7.74	Hindered	Inverted	—
10	-8.10	Available	Inverted	—	-7.51	Available	Correct	Middle	-7.72	Hindered	Inverted	—

Ligand	Xyl$_3$-2AB				Xylotetraose				Cellotetraose			
		Position				Position				Position		
	Binding energy (kcal mol^{-1})	UDP binding site	Non-reducing end	Direction of 4th residue	Binding energy (kcal mol^{-1})	UDP binding site	Non-reducing end	Direction of 4th residue	Binding energy (kcal mol^{-1})	UDP binding site	Non-reducing end	Direction of 4th residue
1	-8.66	Hindered	Slided	—	**-8.25**	**Available**	**Correct**	**Left**	-8.78	Hindered	Inverted	—
2	-8.62	Hindered	Slided	—	-8.15	Hindered	Inverted	—	-8.55	Hindered	Correct	—
3	-8.50	Hindered	Slided	—	-8.09	Available	Inverted	—	-8.52	Hindered	Inverted	—
4	-8.33	Hindered	Inverted	—	-8.08	Hindered	Inverted	—	-8.33	Hindered	Correct	—
5	-8.33	Hindered	Inverted	—	-8.02	Available	Inverted	—	-8.32	Hindered	Correct	—
6	-8.25	Hindered	Inverted	—	-7.86	Hindered	Correct	—	-8.09	Available	Inverted	—
7	**-8.18**	**Available**	**Correct**	**Right**	-7.74	Hindered	Correct	—	-8.05	Hindered	Inverted	—
8	-8.15	Abailable	Inverted	—	-7.53	Available	Correct	Middle	-8.00	Hindered	Inverted	—
9	-8.03	Hindered	Inverted	—	-7.51	Available	Inverted	—	-7.94	Hindered	Inverted	—
10	-8.00	Available	Correct	Left	-7.49	Available	Correct	Left	-7.93	Hindered	Inverted	—

The docking simulation results for various ligands, including Xyl$_3$-2AA, Xyl$_3$-4ABEE, Xyl$_3$-2PA, Xyl$_3$-2AB, xylotetraose, and cellotetraose. Binding energies (kcal mol^{-1}) are listed alongside positional descriptors. Superscripted letters indicate the following statuses of docking: *a*, the ligand interfered with the UDP-binding site; *b*, the ligand in the active site was placed incorrectly as if rolled; *c*, the ligand was placed in an inverted orientation with the non-reducing end positioned outside the active site; *d*, the 4th residue from the non-reducing end positioned at the *left*, *middle*, or *right* sides (see Supplementary Figure S5).

Supplementary Table S3. Interactions between SvIRX10 and ligands (UDP, Xyl_3-2AA, and xylotetraose) in the docking simulations

Ligand/atom		Protein atom	H-bond (Å)	Residues with C-C contact (< 4.4 Å)
**UDP**				
Ribose C2-O		Glu296 OE1	3.1	His267, Tyr271, Pro292
		Tyr272 OH	3.0	
Uridine C2-O		Lys209 NZ	3.1	
Esterified phosphate O^-		His267 NE2	3.1	
		Arg293 NH2	2.9	
Phosphoanhydride O		Arg293 NH1	3.1	
Terminal phosphate double bonded O		Arg248 NH1	3.1	
		Arg293 NH1	3.1	
Terminal phosphate O^-		Arg293 NH1	3.2	
		Arg293 NH2	3.1	
**Xyl_3-2AA**				
X1	ring-O	Trp287 N	3.1	Met75, Asp156, Trp290, Gly286
	C2-O	Asp156 OD1	2.8	
	C4-4	Arg248 NH2	3.0	
X2	C3-O	Asp156 OD2	2.7	His74, Met75, Phe76, Trp287
X3	ring-O	Asp233 OD2	3.1	Leu117, Leu230, Asp233
	C2-O	Ser235 OG	3.1	
2AA	amide-N	Ser235 OG	3.0	Leu117, Ser235, Asn236
	hydroxyl-O	Asn236 OD1	2.9	
	carbonyl-O	Asn236 OD1	3.1	
**Xylotetraose**				
X1	C4-O	Trp290 O	2.9	Ser235, Tyr243, Arg248, Gly286
	ring-O	Trp287 N	3.2	Asp156, Trp287, Trp290
X2	C3-O	Asp156 OD2	2.9	His74, Met75, Phe76, Asp156, Trp287
X3				Leu117, Leu230
X4	C2-O	Thr234 OG1	3.1	Leu230, Ser235
	C3-O	Asp233 OD1	3.1	

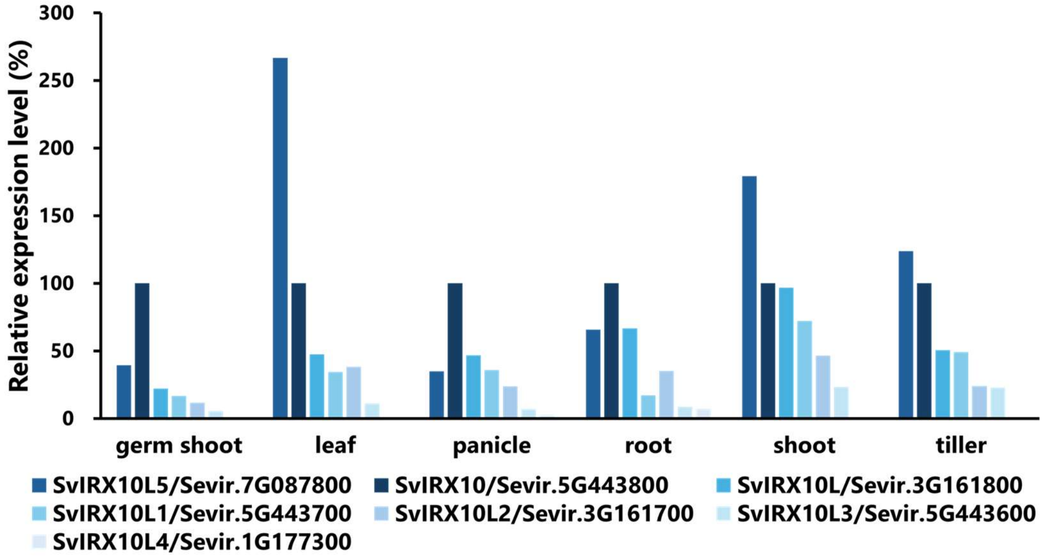

Supplementary Figure S1. Relative gene expression levels of *SvIRX10* homologs in different tissues of *S. viridis.*

The expression values were obtained from the co-expression data section of the GeneAtlas v2 FPKM experiment group of *Setaria viridis* v2.1 (Phytozome genome ID: 500, NCBI taxonomy ID: 4556) in Phytozome v13 [https://phytozome-next.jgi.doe.gov/ (accessed May 20, 2024)]. The expression values of *SvIRX10* were normalized to 100 in each tissue.

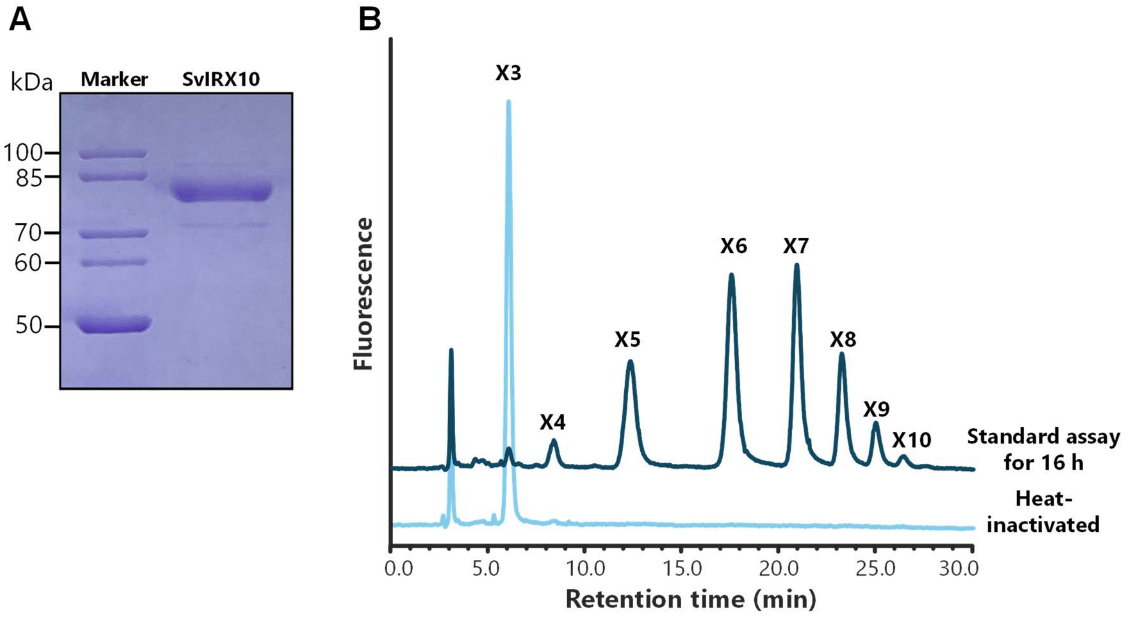

Supplementary Figure S2. Detection of xylan synthase activity by recombinant SvIRX10.

(A) SDS-PAGE and Coomassie Brilliant Blue staining of purified recombinant GFP-fused SvIRX10 (amino acids: 28–415) expressed in Expi293 cells (right lane) and the protein marker (left lane).

(B) Comparison of xylan synthase activity in the standard assay and heat-inactivated enzyme reaction, analyzed by NP-HPLC. The reactions were conducted for 16 h. The numbers of X3 to X10 represent the DP of xylooligomers.

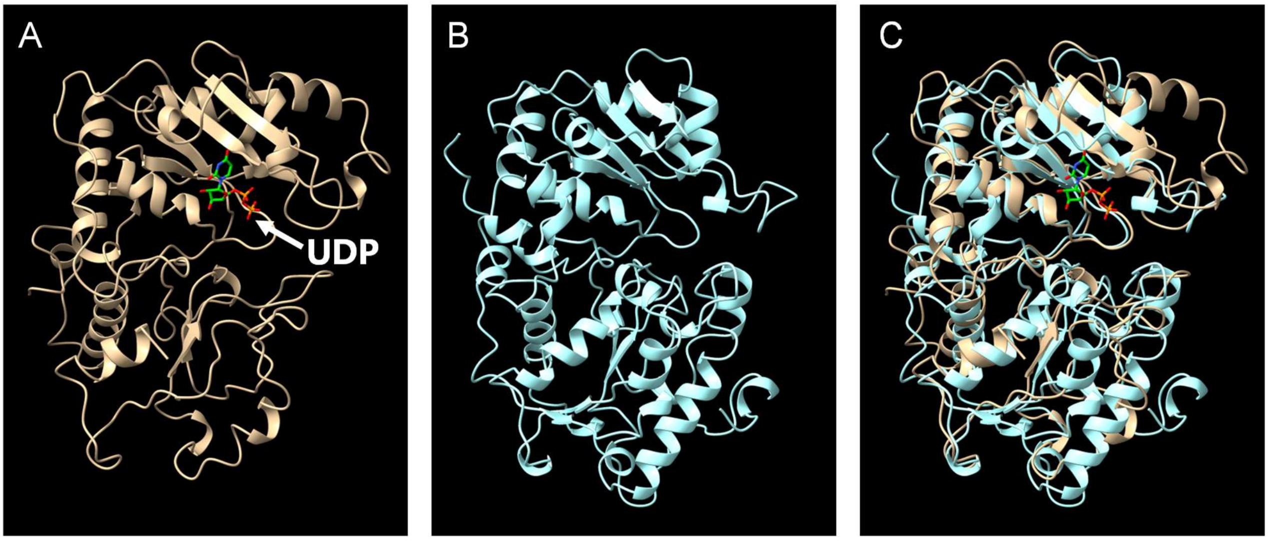

Supplementary Figure S3. Predicted model of the SvIRX10 catalytic domain using ColabFold.

(A) 3D structure of EXT1 GT-B (amino acids: 106–428) in the presence of UDP, obtained from the PDB database entry code 7UQY.

(B) Structural model of SvIRX10 (amino acids: 28–415) predicted by ColabFold.

(C) Structural alignment of SvIRX10 (blue) and EXT1 GT-B (gold), showing similar structures and cleft locations obtained by UCSF ChimeraX. The RMSD between 115 pruned atom pairs was 1.263 Å.

HsEXT1	K Y D Y R E M L H N A T F C L V P R G R R L - - - - - - - G S F R F L **E** A L Q A A C V P V M L S N G
HsEXT2	V F D Y P Q V L Q E A T F C V V L R G A R L - - - - - - - G Q A V L S **D** V L Q A G C V P V V I A D S
HsEXTL3	R E D R L E L L K L S T F A L I I T P G D P R L V I S S G C A T R L F **E** A L E V G A V P V V L G E Q
IRX10/At1g27440	P T T Y Y E D M Q R A I F C L C P L G W A P - - - - - - - W S P R L V **E** A V V F G C I P V I I A D D
IRX10L/At5g61840	P T T Y Y E D M Q R A I F C L C P L G W A P - - - - - - - W S P R L V **E** A V I F G C I P V I I A D D
OsIRX10/Os01g70200	P P T Y Y E D M Q R S V F C L C P L G W A P - - - - - - - W S P R L V **E** A V V F G C I P V I I A D D
OsIRX10L1/Os01g70190	P P T Y Y E D M Q R S I F C L C P L G W A P - - - - - - - W S P R L V **E** A V V F G C I P V I I A D D
OsIRX10L2/Os10g10080	P Q T Y Y E D M Q R A V F C L C P L G W A P - - - - - - - W S P R L V **E** A V V F G C I P V I I A D D
OsIRX10L3/Os01g70180	P A T Y Y E D M Q R A I F C L C P L G W A P - - - - - - - W S P R L V **E** A V V F G C I P V I I A D D
OsIRX10L4/Os02g32110	P A T Y Y E D M Q R S V F C L C P L G W A P - - - - - - - W S P R L V **E** A V V F G C I P V I I A D D
OsIRX10L5/Os04g32670	P P T Y Y E D M Q R A V F C L C P L G W A P - - - - - - - W S P R L V **E** A V V F G C I P V I I A D D
SvIRX10/Sevir.5G443800	P P T Y Y E D M Q R S V F C L C P L G W A P - - - - - - - W S P R L V **E** A V V F G C I P V I I A D D
SvIRX10L/Sevir.3G161800	P P T Y Y E D M Q R S V F C L C P L G W A P - - - - - - - W S P R L V **E** A V V F G C I P V I I A D D
SvIRX10L1/Sevir.5G443700	P P T Y Y E D M Q R A V F C L C P L G W A P - - - - - - - W S P R L V **E** A V V F G C I P V I I A D D
SvIRX10L2/Sevir.3G161700	P P T Y Y E D M Q R S V F C L C P L G W A P - - - - - - - W S P R L V **E** A V V F G C I P V I I A D D
SvIRX10L3/Sevir.5G443600	P S T Y Y E D M Q R A I F C L C P L G W A P - - - - - - - W S P R L V **E** A V V F G C I P V I I A D D
SvIRX10L4/Sevir.1G177300	P A T Y Y E D M Q R A V F C L C P L G W A P - - - - - - - W S P R L V **E** A V V F G C I P V I I A D D
SvIRX10L5/Sevir.7G087800	P A T Y Y E D M Q R A V F C L C P L G W A P - - - - - - - W S P R L V **E** A V V F G C I P V I I A D D

Supplementary Figure S4. Partial sequence alignment of GT47 IRX10 clade members with *Homo sapiens* EXT1, EXT2, and EXTL3.

E349 of EXT1, E296 of SvIRX10, and the conserved E or D in their analogs in GT47 are highlighted in bold.

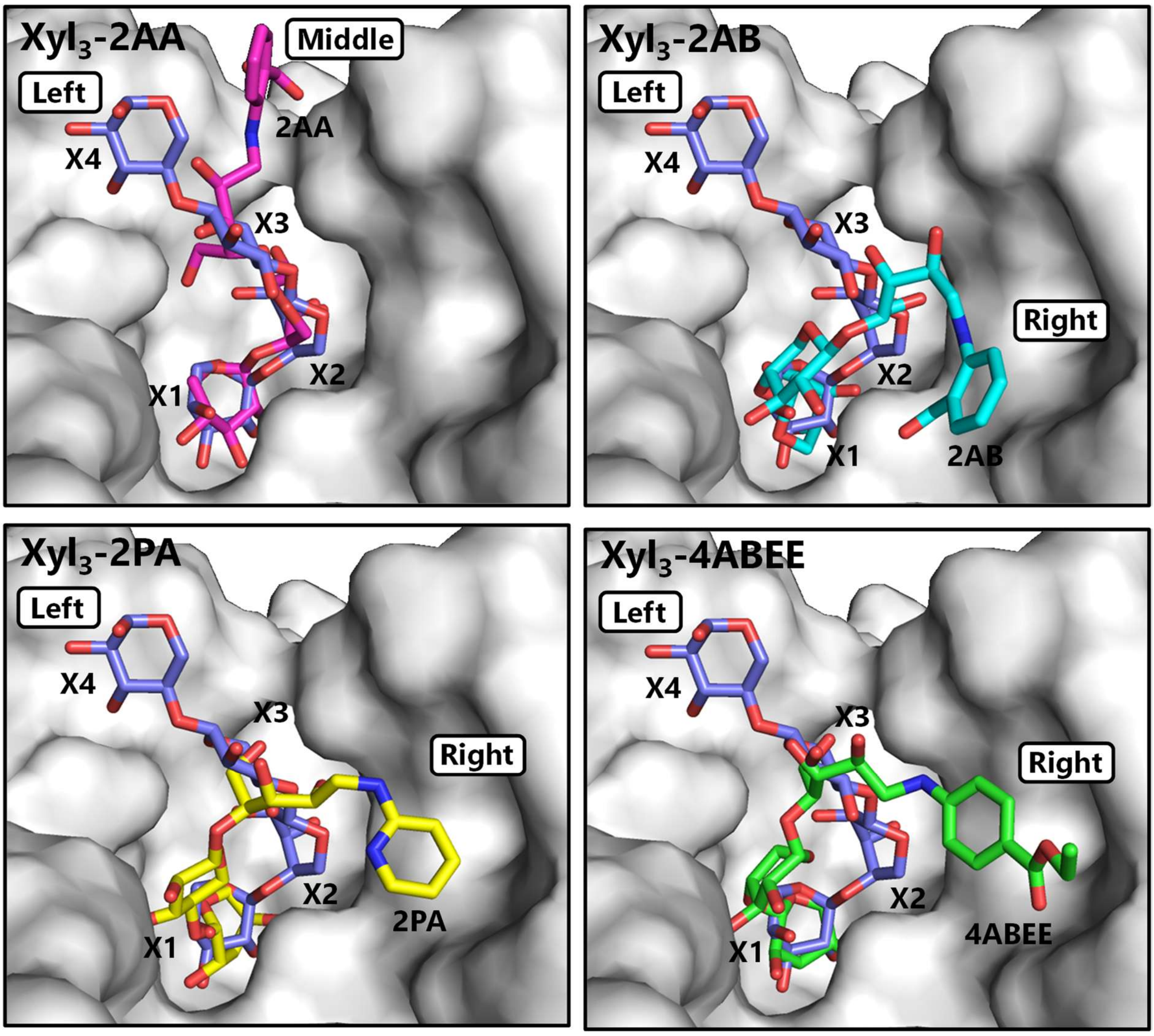

Supplementary Figure S5. Docking simulations of SvIRX10 with fluorescently labeled Xyl_3 and unlabeled xylotetraose.

The parameters utilized in the SwissDock simulations were as follows: the docking method employed was Attracting Cavities 2.0. The box center was defined at coordinates (14 Å, −16 Å, 5 Å), with a box size of 25 Å × 25 Å × 25 Å. The number of Random Initial Configurations (RIC) was also set to 1. Simulations with properly configured ligands and the lowest affinity energy between SvIRX10 and a ligand were visualized, and the molecular surface of SvIRX10 was colored white and gray. The non-reducing end xylosyl residues of fluorescently labeled Xyl_3 and xylotetraose are designated as X1, and the spatial orientation of the 4th residues from the non-reducing end is indicated as *left*, *middle*, or *right* in each simulation. The colors of the carbon atoms in xylotetraose, Xyl_3-2AA, Xyl_3-2AB, Xyl_3-2PA, and Xyl_3-4ABEE are pale blue, magenta, cyan, yellow, and green, respectively.
